# Derivation and validation of the Personal Support Algorithm: an evidence-based framework to inform allocation of personal support services in home and community care

**DOI:** 10.1186/s12913-017-2737-7

**Published:** 2017-11-25

**Authors:** Chi-Ling Joanna Sinn, Aaron Jones, Janet Legge McMullan, Nancy Ackerman, Nancy Curtin-Telegdi, Leslie Eckel, John P. Hirdes

**Affiliations:** 1 School of Public Health and Health Systems, University of Waterloo, 200 University Avenue West, Waterloo, ON N2L 3G1 Canada; 2 Department of Health Research Methods, Evidence, and Impact, McMaster University, 1280 Main Street West, Hamilton, ON L8S 4K1 Canada; 3 Health Shared Services Ontario, (Formerly Ontario Association of Community Care Access Centres), 130 Bloor Street West, Suite 200, Toronto, ON M5S 1N5 Canada; 4 Central Local Health Integration Network, (Formerly Central Community Care Access Centre), 45 Sheppard Avenue East, Suite 700, North York, ON M2N 5W9 Canada

**Keywords:** Home care, Community care, Home support, Health human resources, Decision support, Care planning, Resource allocation, interRAI, RAI-HC, interRAI CHA

## Abstract

**Background:**

Personal support services enable many individuals to stay in their homes, but there are no standard ways to classify need for functional support in home and community care settings. The goal of this project was to develop an evidence-based clinical tool to inform service planning while allowing for flexibility in care coordinator judgment in response to patient and family circumstances.

**Methods:**

The sample included 128,169 Ontario home care patients assessed in 2013 and 25,800 Ontario community support clients assessed between 2014 and 2016. Independent variables were drawn from the Resident Assessment Instrument-Home Care and interRAI Community Health Assessment that are standardised, comprehensive, and fully compatible clinical assessments. Clinical expertise and regression analyses identified candidate variables that were entered into decision tree models. The primary dependent variable was the weekly hours of personal support calculated based on the record of billed services.

**Results:**

The Personal Support Algorithm classified need for personal support into six groups with a 32-fold difference in average billed hours of personal support services between the highest and lowest group. The algorithm explained 30.8% of the variability in billed personal support services. Care coordinators and managers reported that the guidelines based on the algorithm classification were consistent with their clinical judgment and current practice.

**Conclusions:**

The Personal Support Algorithm provides a structured yet flexible decision-support framework that may facilitate a more transparent and equitable approach to the allocation of personal support services.

## Background

Irrespective of age, the vast majority of Canadians living with a chronic health condition or disability prefer to manage their care at home [1, 2]. In Canada, the provinces and territories are responsible for the administration and delivery of home care services, with the exception of federal-level home care programs for veterans, Inuit and on-reserve First Nations communities, and military personnel.

Home care patients are persons who receive home support (e.g., personal support services, homemaking services) or professional services (e.g., nursing, physiotherapy, occupational therapy, social work, speech language pathology, nutritional counselling) in the community. Although the types of services and service delivery models vary across Canada, the provision of personal support services is a key component of all home care programs [3]. Personal support services refer to help with basic self-care tasks such as dressing and bathing known as Activities of Daily Living (ADLs) whereas homemaking services refers to help with more complex skills that enable an individual to live independently in the community known as Instrumental Activities of Daily Living (IADLs). These services are intended to complement the efforts of individuals to live at home safely and maintain acceptable levels of health and functioning with assistance from family, friends, and community resources [2, 3]. Data from the 2009/2010 Home Care Reporting System showed that 42% of home care patients aged 20–64 and 59% of home care patients aged 85 or older received ADL help [1].

In Ontario, publicly funded home care services are coordinated by 14 Local Health Integration Networks (LHINs; formerly known as Community Care Access Centres (CCACs)). A referral for home care services may be initiated by the person requiring help, a family member or friend, or any healthcare professional. The referral is received by a care coordinator who is a regulated health professional and is employed by the LHIN. The care coordinator assesses the person’s needs and potential risks, and develops a care plan based on their clinical judgment as well as the person and family’s unique needs, values, and preferences. In 2014/2015, 27 million hours of personal support were delivered to over 600,000 patients, accounting for 74% of all publicly funded home care services [4].

Under the *Home Care and Community Services Act, 1994, Ontario Regulation 386/99*, a patient can receive up to 120 h of personal support services in the first 30 days of service, and 90 h in any subsequent 30-day period [5]. Patients waiting for placement into a long-term care home, at the end of life, or in “extraordinary circumstances” may exceed these ceilings. Beyond the statutory maximum, however, there is no other provincial standard on the allocation of personal support services. Each LHIN develops their own processes for determining eligibility, priority, and allocation. As a result, a patient with the same degree of need may receive any or no personal support services (or be added to a waitlist) and may receive more or fewer hours based on the LHIN in which he or she lives.

In July 2014, amendments to Regulation 386/99 came into effect that enabled agencies other than LHINs, namely community support service (CSS) agencies, to provide personal support services for low acuity patients. As Ontario moves toward a collaborative home and community-based care coordination model, it is imperative that LHINs and CSS agencies develop standard assessment practices and consistent service priorities and levels within and across agencies so that access to personal support services is based on a person’s need and not the access point [6, 7]. Transparent and equitable allocation processes, locally adapted models, and quality monitoring have also been identified as essential elements of a collaborative personal support services delivery model [6, 7].

Given the importance of personal support services for helping many individuals to stay in their homes, and in the absence of province-wide policies, a provincial working group was established to develop evidence-based clinical tools to inform service planning and to promote provincially consistent practice patterns. Working group members included clinical leads from seven LHINs; staff from the Information Management, Education Services, and Client Services teams of Health Shared Services Ontario (HSSOntario; formerly the Ontario Association of Community Care Access Centres (OACCAC)); and researchers from the University of Waterloo/interRAI Canada.

This study reports on the derivation, validation, and testing of a decision support algorithm to differentiate need for personal support services, as well as a novel way to present personal support allocation guidelines based on the algorithm classification.

## Methods

### Study design and sample

Two population cohorts — a home care cohort and a community support cohort — were created. The home care cohort was created by prospectively linking home care clinical assessment records to an administrative record of billed services. The cohort represented all adult home care patients in Ontario’s LHINs who were assessed between January and December 2013 and expected to be on service for at least 60 days (i.e., long-stay patients). If a patient had multiple assessments, the assessment closest to the mid-year was selected. Patients who were assessed in hospital, received case management or long-term care placement services only, or received less than three weeks of active service were excluded. These exclusions helped to focus on community-living home care patients requiring long-term home care services to maintain independence or delay institutionalisation. Patients above the 99th percentile of personal support utilisation were also excluded since their exceptional individual circumstances, rather than clinical characteristics, may more readily explain differences in the allocation of hours close to or above the statutory maximum. The community support cohort consisted of the initial clinical assessment record for unique clients assessed between 2014 and 2016 from CSS agencies in Ontario. This cohort represented only CSS agencies that routinely upload their assessments to the Integrated Assessment Record (IAR). This may have excluded agencies that are not required to complete a standardised assessment if their services are limited to non-clinical supports and agencies that do not voluntarily upload their assessments to the IAR. In total, the final sample included 128,169 home care patients and 25,800 community support clients. It is possible that some persons were counted in both cohorts; however, in practice, persons receiving both home care services and community support services are assessed with the home care instrument only.

These records are maintained by the HSSOntario and individual CSS agencies and are sent in de-identified, linkable form to a secure data server at the University of Waterloo. Ethics approval was obtained from the University of Waterloo’s Office of Research Ethics (ORE# 18228 and 19917).

### Independent variables

The independent variables are drawn from standardised comprehensive clinical assessments developed by interRAI, an international not-for-profit group of researchers that aims to improve care for vulnerable persons through the use of comprehensive assessment systems across sectors and countries [8]. The Resident Assessment Instrument-Home Care (RAI-HC) is used with all adult, non-palliative, long-stay home care patients in Ontario [3]. The interRAI Community Health Assessment (CHA) consists of a core assessment and four types of supplements, including the Functional Supplement, and has been adopted by various CSS agencies in Ontario. In terms of content overlap, the RAI-HC and core CHA cover the same domains although the core CHA has fewer questions. Completion of the core CHA and the Functional Supplement is basically equivalent to completing the RAI-HC. Assessors are health professionals trained in the administration of the interRAI assessment and the use of its embedded clinical scales and algorithms. Assessors administer the assessment on admission and every 6 to 12 months, or sooner in the case of a significant change in health status. The reliability and validity of the RAI-HC instrument is well-documented in the literature [8–11]. A recent study examining data collected using the RAI-HC and interRAI CHA in Ontario and British Columbia found evidence of good data quality, and endorsed the use of this data for supporting clinical practice and policy decisions in the two sectors [12].

Candidate variables were identified based on a list of variables agreed upon by the clinical members of the working group and a series of regression analyses adjusted for LHIN. These variables included ADL and cognitive impairment, incontinence, falls, activity level and balance, and caregiver availability and distress.

### Dependent variables

Need for personal support was operationalised through historical service allocation patterns. In other words, care coordinators apply their clinical expertise to interpret objective and subjective cues of need and their allocation decisions reflect their judgment about the patient’s level of need and risk in relation to other patients and in the context of available resources.

The weekly hours of personal support based on the record of billed services was the primary dependent variable. For each patient, all services that were identified as “personal support” occurring within 12 weeks of the RAI-HC assessment date were retrieved. To calculate a weekly mean, the total number of hours was divided by the number of service days (i.e., difference in days between first and last visit) and multiplied by 7; thus, the denominator was adjusted for patients who did not receive service for the full observation period.

A secondary dependent variable was defined as the number of hours of personal support reported on the clinical assessment. As part of the assessment process, assessors ask patients and their caregivers to estimate the patient’s receipt of formal care in the last seven days, rounded to the nearest 10 min. In this study, the time documented under “home health aides” and “homemaking services” was summed.

There are important distinctions between the two dependent variables. Billed services account for LHIN-provided services only whereas estimated services noted in the interRAI assessment cover personal support received from any source that may have been paid by the public system, private insurance, or out-of-pocket. High accuracy of the billed services record is maintained by counteracting incentives in which service providers seek to be remunerated for every home visit and LHINs wish to pay for visits that actually occurred. In contrast, the secondary dependent variable is subject to recall bias, but covers all personal support services received irrespective of source. The time periods also differ. Billed services capture the services received 12 weeks after the assessment date while estimated services reflect a 7-day lookback period preceding the assessment.

### Algorithm derivation

The population cohorts were randomly partitioned for algorithm derivation (70%) and validation (30%). SAS Enterprise Miner 13.1 was used to perform decision tree analysis [SAS Institute Inc., Cary, NC]. Decision tree analysis is a recursive partitioning method that depicts a set of decision rules as nodes and branches. The tree structure produces terminal nodes whose members are as homogeneous as possible within the same node and as distinct as possible from members of other nodes. Decision tree analysis offers a complementary perspective to regression methods that are typically limited to linear effects and two-way interactions. In contrast, recursive partitioning can work with higher order interactions, “asymmetric” interactions (i.e., different cut-offs on the same variable), and can be visually represented to aid the interpretation of complex interactions [13]. Decision trees can be built according to standard algorithms, or in the case of SAS Enterprise Miner, the researcher can specify the parameters directly. The key parameters chosen in this study were: (1) F-test (i.e., variance reduction) as the splitting criterion; (2) binary (preferred) and multi-way splitting at each node; (3) a maximum number of six levels in the tree structure; (4) a minimum number of 100 observations in any terminal node; and (5) Bonferroni adjustments applied on every split. If the *p*-values had not been corrected, there would be a high probability of a false positive F-test (i.e., type I error) because of the vast number of candidate variables and possible cut-off points of variables [14]. The target variable was the mean weekly hours of personal support based on the record of billed services. The number of hours of personal support reported on the interRAI CHA was used as a secondary target to test the relevance of the models in the community support sector.

Approximately 20 tree structures were developed through interactive training. Growth of the trees and selection of the final tree was guided by the clinical expertise of the working group and the ranking of variable importance provided by Enterprise Miner. Different conceptual trees were tested by entering both strong predictors of personal support and known covariates (e.g., age) as the first variable. Multiple candidate variables and cut-off values were considered at each split. As a result of developing the trees manually (rather than automatically), the working group gained valuable insight into the combinations of variables representing frequently occurring (i.e., expected) groups as well as rare groups with distinct personal support utilisation patterns. Each subsequent tree structure was informed by the accumulation of knowledge from previous cycles of developing and discussing trees.

### Algorithm validation

Decision trees are susceptible to over-fitting with respect to the derivation dataset if the phenomenon is not generalisable to other data. The purpose of the validation dataset was to avoid over-fitting by checking for unstable values of the target variable in an independent sample. The mean and median weekly hours of personal support were calculated for each terminal node. Nodes with similar averages were combined into higher-level groups that would be used for clinical decision support. The decision tree was coded in SAS 9.4 for further validation and external testing [SAS Institute Inc., Cary, NC].

Candidate trees were evaluated for statistically and clinically significant ability to classify need for personal support. Goodness of fit was examined through explained variance (or coefficient of determination) that evaluates the proportion of the variability in the actual hours explained by the model, and the coefficient of variation that evaluates the relative closeness of the predictions to the actual hours. The ability to differentiate groups was reported as a ratio of the highest group mean to the lowest group mean. Clinical significance was evaluated based on the clinical recognisability and relevance of each combination of variables. In addition, the performance of the final decision tree was evaluated for consistency across LHINs as well as over time using provincial home care data from calendar years 2011 and 2012.

## Results

### Sample characteristics

The population cohorts are described in Table 1. Persons receiving home care and community support services were mostly older and female. The mean age was 78.0 ± 14.2 years among home care patients and 77.4 ± 14.0 years among community support clients. In general, home care patients were more likely to demonstrate functional and cognitive impairment, bladder incontinence, recent falls, unstable health conditions, and poor self-reported health. Persons whose needs were served mainly through CSS agencies were more likely to live alone and report psychiatric or mood conditions. Shortness of breath and dementia occurred at comparable rates in both cohorts.

**Table 1:** Demographic, clinical, and service utilisation characteristics of the home care cohort and community support cohort

% (n)			Home care cohort (*n* = 128,169)	Community support cohort (*n* = 25,800)	*p*-value, home care vs community support	Home care patients receiving any personal support services (*n* = 107,407)
Age						
65 or older			83.8 (107,359)	84.2 (21,714)	0.11	86.8 (93,204)
85 or older			36.8 (47,157)	33.5 (8653)	<0.001	39.9 (42,874)
Female			64.9 (83,211)	66.0 (17,035)	<0.001	67.2 (72,216)
Married			38.0 (48,662)	28.2 (7284)	<0.001	36.4 (39,098)
Living alone			33.1 (42,453)	56.0 (14,454)	<0.001	33.5 (35,947)
Instrumental Activities of Daily Living Difficulty Scale	Minimal difficulty	0–1	5.2 (6607)	26.7 (6893)	<0.001	2.9 (3090)
	Moderate difficulty	2–4	30.1 (38,603)	30.0 (7747)		28.0 (30,054)
	Great difficulty	5–6	64.7 (82,959)	43.3 (11,160)		69.1 (74,263)
Activities of Daily Living Hierarchy	Independent	0	49.3 (63,240)	44.3 (11,418)	<0.001	44.7 (47,977)
	Mostly independent	1–2	32.3 (41,431)	16.4 (4219)		35.4 (38,058)
	Extensive assistance	3–4	13.8 (17,700)	10.4 (2685)		15.1 (16,178)
	Mostly dependent	5–6	4.5 (5798)	3.7 (945)		4.8 (5194)
	Missing		–	25.3 (6533)		–
Cognitive Performance Scale	No impairment	0	30.4 (38,969)	45.7 (11,801)	<0.001	26.4 (28,403)
	Mild impairment	1–2	55.3 (70,909)	43.8 (11,297)		57.6 (61,835)
	Moderate impairment	3–4	9.3 (11,912)	7.4 (1908)		10.4 (11,213)
	Severe impairment	5–6	5.0 (6379)	3.1 (794)		5.6 (5956)
Functional decline in last 90 days			51.2 (65,576)	21.8 (5622)	<0.001	52.6 (56,497)
Cognitive decline in last 90 days			18.5 (23,665)	15.8 (4073)	<0.001	19.9 (21,417)
Bladder incontinence, at least weekly episode			53.5 (68,582)	33.2 (8567)	<0.001	58.6 (62,942)
Fall in last 90 days			37.5 (48,119)	24.9 (6426)	<0.001	38.3 (41,103)
Unsteady gait			74.9 (95,936)	52.6 (13,580)	<0.001	78.1 (83,904)
Shortness of breath			31.3 (40,062)	32.1 (8290)	0.006	31.6 (33,988)
Conditions or diseases that make cognition, ADL, mood, or behaviour patterns unstable			49.3 (63,197)	31.3 (8062)	<0.0001	50.8 (54,515)
Poor self-reported health			23.6 (30,235)	13.4 (3447)	<0.0001	23.8 (25,505)
Diagnosed conditions	Cardiovascular conditions		42.7 (54,720)	33.9 (8741)	<0.0001	44.6 (47,940)
	Dementia		20.4 (26,081)	20.2 (5223)	0.70	22.3 (23,929)
	Psychiatric or mood conditions		18.5 (23,743)	28.0 (7216)	<0.0001	18.7 (20,072)
Received any personal support services			83.8 (107,407)	22.3 (5758)	<0.0001	100.0 (107,407)

Over four-fifths, or 83.8%, of the total home care sample received any personal support services within 12 weeks of the assessment. The mean hours of billed (i.e., LHIN-provided) personal support received by the total sample was 4.13 ± 4.85 h per week (Table 2). The median weekly hours was 2.15 h and the interquartile range was 1.00 to 5.75 h. Among patients who received any billed personal support services, the mean increased to 4.93 ± 4.91 h per week and the median increased to 2.95 h per week. On average, the mean hours of personal support received from all sources was two hours greater than the mean hours of LHIN-provided services.

**Table 2:** Amount of personal support services received in the home care cohort and community support cohort

	Home care cohort (*n* = 128,169)	Home care patients receiving any personal support services (*n *= 107,407)	Community support cohort (*n* = 25,800)
Mean weekly hours of personal support services, billed^a^	4.13 ± 4.85	4.93 ± 4.91	N/A
Mean weekly hours of personal support services, estimated^b^	6.13 ± 13.6	6.72 ± 13.4	1.32 ± 8.98

^a^Calculated from the billed services record and represents services received from LHIN

^b^Calculated from the interRAI assessment and represents services received from any source

In contrast, only 22.3% of the total community support cohort received any personal support services. The mean hours of personal support received from all sources was 1.32 ± 8.98 h per week. Among community support clients receiving any personal support services, the median weekly hours was 6.00 h and the interquartile range was 2.75 to 12.33 h.

### Personal Support Algorithm derivation

**Fig. 1:**
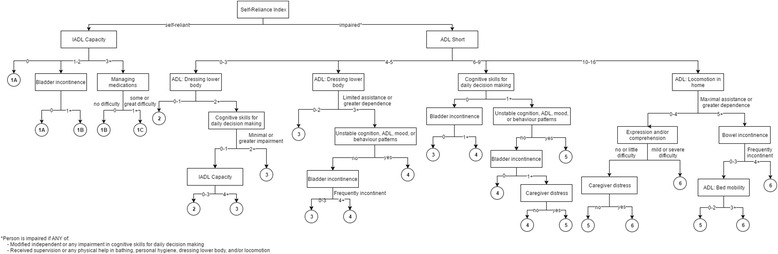
The Personal Support Algorithm

The decision tree consisted of 25 terminal nodes that were collapsed into six groups and ordered hierarchically such that a higher group represents greater need for personal support (Fig. 1). The root node was divided on the Self-Reliance Index, a dichotomous indicator of cognitive and physical impairment. Patients are assessed as impaired if they have any difficulty making safe and reasonable decisions or if they require supervision or any physical help with bathing, personal hygiene, dressing lower body, or walking (or wheeling) indoors or outdoors. For patients who were impaired in the Self-Reliance Index, the 7-item ADL Long Scale and the 4-item ADL Short Scale showed similar discriminatory power [15]. Therefore, the shorter scale was selected as the second variable to minimise the required number of assessment items. Among those who scored low on the ADL Short Scale (i.e., low ADL impairment) and needed supervision or any physical help with dressing upper body, the presence of either some impairment in daily decision making or great difficulty with instrumental activities of daily living (IADLs) was associated with greater need for personal support. For patients with moderate to high ADL impairment, the presence of cognitive impairment, unstable health patterns, and bladder incontinence differentiated need for personal support. Combinations of late loss ADLs and bowel incontinence or communication difficulties and caregiver distress differentiated patients at the highest level of ADL impairment. Among patients who did not trigger the Self-Reliance Index, difficulty with IADLs helped to further differentiate Group 1 into three levels (1A, 1B, 1C).

**Fig. 2:**
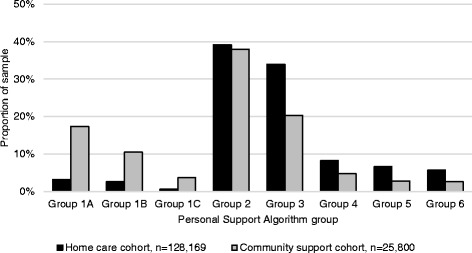
Distribution of algorithm groups across the home care cohort and community support cohort

The distribution of algorithm groups between the cohorts was notable (Fig. 2). Over one-third of both cohorts were in Group 2, indicating substantial overlap in the level of need across populations. The next largest group was Group 3 in the home care cohort and Group 1 in the community support cohort. One in five patients in the RAI-HC-assessed cohort were in Groups 4, 5, or 6 compared to one in ten interRAI CHA-assessed community support clients.

Additional attempts were made to differentiate Group 2, but further splitting neither significantly improved the algorithm’s performance nor changed the group distribution by more than 2%. In Table 3, the interquartile range of personal support hours received by Group 2 was less than two hours and was much narrower than other groups, suggesting need for personal support within Group 2 is quite homogeneous.

**Table 3:** Group means and percentile distributions of billed hours of personal support across Personal Support Algorithm groups

Personal Support Algorithm group	% (n)	Mean ± standard deviation	Median (Q_1_–Q_3_)	Coefficient of variation
1	6.4 (8154)	0.4 ± 1.4	0.0 (0.0–0.0)	3.89
2	45.5 (58,807)	2.3 ± 2.7	1.7 (0.9–2.8)	1.18
3	28.2 (36,130)	4.8 ± 4.3	3.4 (1.9–6.7)	0.91
4	7.6 (9623)	6.9 ± 5.6	5.7 (2.7–10.2)	0.81
5	6.6 (8523)	8.4 ± 6.2	7.0 (3.5–13.1)	0.74
6	5.7 (7432)	11.2 ± 6.8	12.0 (6.3–14.8)	0.60

### Personal Support Algorithm validation

Each of the six groups had significantly different means and distinct percentile distributions (Table 3). The mean weekly hours of personal support received was 0.4 ± 1.4 h in Group 1 and increased 32-fold to 11.2 ± 6.8 h in Group 6. With each increase in group, the percentile distribution also shifted rightward as depicted by the interquartile range. In the derivation dataset, the algorithm explained 30.8% of the variability in billed personal support hours (Table 4). At the LHIN-level, the explained variance ranged from 25.7% to 41.8% and the coefficient of variation ranged from 81.7 to 112.8. The ratio of the size of the highest to the lowest group means varied from a 15-fold to 84-fold difference. There was remarkable consistency in these measures when the Personal Support Algorithm was fit to home care cohorts from previous years, achieving values of explained variance of 32.2% in the 2012 dataset and 31.4% in the 2011 dataset.

**Table 4:** Performance of the Personal Support Algorithm in the derivation and validation datasets

	Explained variance	Coefficient of variation	Ratio of Group 6 to Group 1 means
Derivation dataset			
2013 (all LHINs)	30.8%	97.6	32.1
Stratified by LHIN	25.7%–41.8%	81.7–112.8	14.6–84.1
Validation datasets (in previous years)			
2012 (all LHINs)	32.2%	98.6	39.2
2011 (all LHINs)	31.4%	99.7	36.3

### Guideline development

The Personal Support Algorithm was applied to all adult long-stay home care patients who were admitted and received a RAI-HC assessment between April 2014 and March 2015. In practice, a patient’s care plan may not be followed as intended by the care coordinator. For example, the patient may be admitted into hospital that reduces the number of billed hours during the observation period, but does not reflect any lesser need for personal support. To better represent true service allocations, the following populations were excluded: (1) patients residing in a retirement home, supportive housing, or assisted living; (2) patients who were on a waitlist or on hold and did not receive any personal support in the 12-week period; and (3) patients who were in Groups 3 to 6 but received no personal support. This last group of patients likely received no personal support for reasons other than absence of need (e.g., patient or family declined offered services). Unlike the derivation and validation datasets, patients at the highest level of personal support utilisation were not excluded to reflect the real-world applicability of the algorithm.

**Fig. 3:**
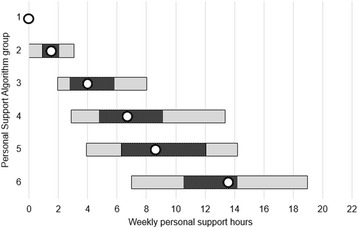
Conceptual framework for allocation guidelines. The median is represented by the dot. The 35th and 65th percentile and 20th and 80th percentile are represented by the patterned bar and solid bar, respectively

Next, the distributions of hours were retrieved for each group. These percentiles were used to create the conceptual framework shown in Fig. 3. The median (50th percentile) is represented by the dot. The patterned bar is bounded by the 35th and 65th percentile. The solid bar is bounded by the 20th and 80th percentile. For a given patient in a certain group, the ranges convey the frequency at which care coordinators expect to allocate personal support services. Allocations are expected to be made around the median most frequently in the patterned bar, occasionally in the solid bar, and only in exceptional cases beyond the solid bar. The intent of the framework is to encourage the allocation of personal support toward a central value, resulting in allocation decisions that approximate a normal distribution.

### Pilot testing

A pilot test of the Personal Support Algorithm was conducted to gauge user acceptance and identify areas for potential improvement. The pilot test ran from June to July 2015 with 28 care coordinators across six LHINs. Participating LHINs were chosen to reflect the diversity of urban and rural geographies as well as variation in care coordinator practices. At each pilot site, a manager was designated to support the pilot test and a minimum of three care coordinators with at least three months of home care experience were asked to complete 10 to 15 episodes of care. All care coordinators and managers participated in an information and training session via webinar hosted by HSSOntario and University of Waterloo/interRAI Canada prior to the pilot. For each episode of care, care coordinators completed a RAI-HC assessment and followed their usual practice to determine the patient care plan and allocation of personal support hours. Within 24 h of completing the patient’s care plan, the care coordinator received an online survey that displayed the Personal Support Algorithm group, the suggested hours for that group (i.e., lower bound, median, and upper bound), and survey questions. The initial goal of 30 assessments per LHIN was increased to 45 due to strong interest in the study, resulting in 276 completed assessments. All data collection and analysis was done internally by HSSOntario.

The pilot test’s primary objective was to measure user acceptance that was chiefly assessed by asking the care coordinator to reflect on whether the suggested hours were clinically appropriate in meeting their patient’s needs. Care coordinators provided an affirmative answer in 257 of 276 cases (93.1%). This percentage ranged from 87% to 100% depending on the individual LHIN. Even though the care coordinators were blinded to the algorithm results, their actual allocation of hours fell within the suggested hours in 246 cases (89.1%). Functional complexity, grandfathered service levels, and the provision of medical or meal reminders were the most common reasons for exceeding the upper bound. Personal preference or having a private pay caregiver, access to adequate community supports, and residing in a retirement home were the most common reasons for allocating fewer than the suggested hours.

Focus groups with care coordinators and managers who participated in the pilot test were held via teleconference within a month of study completion. The focus group questions were designed to elaborate on care coordinators’ and managers’ experience with the guidelines, identify strategies to address training needs, and identify opportunities or strategies to support user acceptance of the algorithm and guidelines. In general, the pilot test participants thought the algorithm could promote provincial consistency in personal support service allocation and believed the guidelines aligned well with their clinical decision-making processes as well as current practice. Exceptions such as patients receiving enhanced services as part of special programs were identified; however, it was agreed that clear processes and managerial support on requesting exceptions were needed rather than changes to the algorithm or guidelines. Participants thought that care coordinators should be knowledgeable about the variables within the decision tree and reiterated the importance of accurate coding. Of principal concern was the ability of all LHINs to offer services at the same median level because of varying budgetary constraints.

## Discussion

The Personal Support Algorithm differentiates need for personal support services and provides a structured yet flexible decision-support approach for allocating hours of personal support. The framework can allow for population-level equity in the allocation of personal support services while providing for individual-level flexibility in the person-level decisions made by care coordinators. Regional variations in the median allocation of personal support services within the Personal Support Algorithm groups can be used to determine whether non-patient factors are affecting allocations across geographic regions. At the same time, care coordinators can adjust person-level allocations to take into account the needs, strengths, and preferences not built into the algorithm. Software systems can be used to provide care coordinators with dynamic feedback on their allocation patterns over time and compared to peers within and outside of their region. Hence, care coordinators can be flexible on a case-by-case basis while striving for an equitable approach in their overall caseload compared with other clinicians.

As expected, measures of ADL and cognitive impairment were prominent drivers of receipt of personal support. Other factors such as difficulty with IADLs, bladder and bowel incontinence, unstable patterns, difficulty with communication, and caregiver distress were also important. In a systematic review of literature on care coordinators’ resource allocation decisions, a patient’s clinical characteristics were found to have the greatest influence in resource allocation decision-making [16]. Previous studies have also explored the role of patient preferences, existing receipt of formal and informal care, personal resources, case manager background and experience, and information- and system-related factors on decisions [16–20]. But while the typical factors considered in the decision-making process may be known, the complexities of each patient and family’s unique goals and circumstances may require adjustment to the priorities or approaches to care [21]. Corazzini [22] found that care coordinators exercised discretion in the application of policies, especially in the presence of factors that were not captured in standardised assessments. In an environment characterised by high uncertainty, Giacomini and colleagues suggested that tensions between values are better reconciled through “narrative or juridical forms of reasoning and judgment” [23]. The framework proposed by this study attempts to bring together patient characteristics that represent need while acknowledging the host of other factors related to the patient, family, and health care system that cannot be known predictably. The algorithm stratifies patients based on major indicators of need and suggests a range of hours that will likely address the needs of a “typical” patient. However, the actual allocation of hours depends on the consideration of other information sources and is under the control of the care coordinator. Rather than constrain decision-making as in the traditional view of guidelines, the framework standardises the magnitude of the care coordinator’s response so that the relative and absolute allocation of personal support services across Ontario can be consistent and equitable.

The Personal Support Algorithm explains 30.8% of the variance in personal support allocation, demonstrating good performance in relation to existing predictive models in home care. The prediction of health service allocation or cost is rarely easy, especially of health services delivered in the community. In comparison, the explained variance of other home care case mix systems ranges from 17% to 33% [24–29]. In Ontario, the Resource Utilisation Groups for Home Care (RUG-IIII/HC) system that is used to inform home care resource planning and funding explains 37.3% variance in total home care resource use but only explains 21.4% of the variance in personal support service alone [28]. Further, the Personal Support Algorithm has high discriminatory ability as demonstrated by the 32-fold difference between the highest and lowest group means.

It should be noted that the selection of percentile values to define the ranges in the conceptual framework may be adjusted. For illustrative purposes, the 35th to 65th percentile range was chosen to capture the 30% of cases distributed around the median. If preferred by policy makers, these target allocations could be narrowed to the 45th to 55th percentiles. This narrower range would reduce overlapping hours between groups when applied to practice, but would also reduce clinician autonomy in considering other contextual factors that may determine the most appropriate case-by-case allocations (e.g., availability of family supports).

The strengths of this study are largely attributable to the interdisciplinary and collaborative nature of the working group. Existing data agreements granted access to a large database of clinical and administrative data that is representative of the home care and community support populations in Ontario. The clinical members of the working group increased the clinical relevance and utility of the project outputs in many ways, including the identification of candidate variables, growth of the decision trees, and selection of the final tree. HSSOntario provided the infrastructure, information management personnel, and educational resources to run the pilot and analyse the results. While many studies of case management decision-making rely on hypothetical vignettes that are usually unable to capture the complexities of real cases, this study evaluated the utility of the new guidelines for actual patients while care coordinators remained blinded to the algorithm results.

The Personal Support Algorithm as it is currently calculated from the RAI-HC and interRAI CHA is limited to long-stay home care patients. Additional testing and potential refinement is needed for short-stay, palliative, or post-acute home care populations using either the same instruments or instruments from the interRAI suite (e.g., interRAI Contact Assessment, interRAI Palliative Care). Validation of the algorithm in the community support sector could be strengthened by using a more accurate source of personal support utilisation data than the hours recorded on the interRAI CHA assessment. Lastly, professionals applying this tool will need to be mindful that the Personal Support Algorithm explains about 31% of the variance in personal support hours. The algorithm integrates information on some of the most predictive and common indicators of need for personal support observed at the population level. It can be used to inform the decision-making process, but care coordinators should consider other contextual factors (especially social and economic environments) when estimating the specific needs of individual patients.

Soon after the pilot test results were disseminated, functionality to calculate the Personal Support Algorithm was implemented into the Client Health and Related Information System (CHRIS) that is a web-based patient management system used by the LHINs. This technology solution will enable LHINs to view the provincial baseline of the amount of personal support authorised for patients with similar needs and to track and compare service allocation patterns across health regions. The proportion of patients receiving services within the expected provincial ranges could be used as a quality indicator. Implementation of the algorithm and guidelines would enable research into the relationship between the intensity of personal support services and patient outcomes that could be used, in turn, for updating the provincial ranges. With the larger home and community-based care coordination model and the principles of transparency and equity in mind, a future study could evaluate optimal ways to translate the algorithm and/or guidelines for patients and families, care coordinators, and other health professionals.

The development of the Personal Support Algorithm in the Ontario context is also relevant for home care systems in other countries that have adopted the interRAI suite of instruments. To test the generalisability of the algorithm on an international scale, this study’s authors are collaborating with researchers from a cross-European project (Identifying best practices for care-dependent elderly by Benchmarking Costs and outcomes of community care (IBenC)) to compare the level of formal and informal home support use in Canada and six European countries. Attitudes toward formal and informal care vary widely by country, and similarly, each home care system is unique in its structural characteristics, service delivery models, funding, and care processes. For instance, in Italy, approximately one-sixth of the population provides care to someone with high functional impairment compared to one in nine in Germany [30]. A more concrete understanding of need for functional support and the ability to compare cost and quality indicators will optimise the design of home and community support systems to better meet the needs of persons managing their care at home.

## Conclusions

The Personal Support Algorithm provides a structured yet flexible decision-support framework that may facilitate a more transparent and equitable approach to the allocation of the personal support services in Ontario.

## Abbreviations

ADL: Activity of Daily Living
CCAC: Community Care Access Centre
CHA: Community Health Assessment
CHRIS: Client Health and Related Information System
CSS: Community Support Service
HSSOntario: Health Shared Services Ontario
IADL: Instrumental Activity of Daily Living
IAR: Integrated Assessment Record
IBenC: Identifying best practices for care-dependent elderly by Benchmarking Costs and outcomes of community care
LHIN: Local Health Integration Network
OACCAC: Ontario Association of Community Care Access Centres
RAI-HC: Resident Assessment Instrument-Home Care
RUG-III/HC: Resource Utilisation Groups for Home Care
